# Involvement of *DPP9* in gene fusions in serous ovarian carcinoma

**DOI:** 10.1186/s12885-017-3625-6

**Published:** 2017-09-11

**Authors:** Marianne Lislerud Smebye, Antonio Agostini, Bjarne Johannessen, Jim Thorsen, Ben Davidson, Claes Göran Tropé, Sverre Heim, Rolf Inge Skotheim, Francesca Micci

**Affiliations:** 1 Section for Cancer Cytogenetics, Institute for Cancer Genetics and Informatics, The Norwegian Radium Hospital, Oslo University Hospital, Oslo, Norway; 2 Centre for Cancer Biomedicine, University of Oslo, Oslo, Norway; 3 Department of Molecular Oncology, Institute for Cancer Research, The Norwegian Radium Hospital, Oslo University Hospital, Oslo, Norway; 4 Department of Pathology, The Norwegian Radium Hospital, Oslo University Hospital, Oslo, Norway; 5 Faculty of Medicine, University of Oslo, Oslo, Norway; 6 Department of Gynecology, The Norwegian Radium Hospital, Oslo University Hospital, Oslo, Norway

**Keywords:** Ovarian carcinoma, Fusion genes, Gene expression, DPP9

## Abstract

**Background:**

A fusion gene is a hybrid gene consisting of parts from two previously independent genes. Chromosomal rearrangements leading to gene breakage are frequent in high-grade serous ovarian carcinomas and have been reported as a common mechanism for inactivating tumor suppressor genes. However, no fusion genes have been repeatedly reported to be recurrent driver events in ovarian carcinogenesis. We combined genomic and transcriptomic information to identify novel fusion gene candidates and aberrantly expressed genes in ovarian carcinomas.

**Methods:**

Examined were 19 previously karyotyped ovarian carcinomas (18 of the serous histotype and one undifferentiated). First, karyotypic aberrations were compared to fusion gene candidates identified by RNA sequencing (RNA-seq). In addition, we used exon-level gene expression microarrays as a screening tool to identify aberrantly expressed genes possibly involved in gene fusion events, and compared the findings to the RNA-seq data.

**Results:**

We found a *DPP9*-*PPP6R3* fusion transcript in one tumor showing a matching genomic 11;19-translocation. Another tumor had a rearrangement of *DPP9* with *PLIN3*. Both rearrangements were associated with diminished expression of the 3′ end of *DPP9* corresponding to the breakpoints identified by RNA-seq. For the exon-level expression analysis, candidate fusion partner genes were ranked according to deviating expression compared to the median of the sample set. The results were collated with data obtained from the RNA-seq analysis. Several fusion candidates were identified, among them *TMEM123*-*MMP27*, *ZBTB46*-*WFDC13*, and *PLXNB1-PRKAR2A*, all of which led to stronger expression of the 3′ genes. In view of our previous findings of nonrandom rearrangements of chromosome 19 in this cancer type, particular emphasis was given to changes of this chromosome and a *DDA1*-*FAM129C* fusion event was identified.

**Conclusions:**

We have identified novel fusion gene candidates in high-grade serous ovarian carcinoma. *DPP9* was involved in two different fusion transcripts that both resulted in deregulated expression of the 3′ end of the transcript and thus possible loss of the active domains in the DPP9 protein. The identified rearrangements might play a role in tumorigenesis or tumor progression.

**Electronic supplementary material:**

The online version of this article (10.1186/s12885-017-3625-6) contains supplementary material, which is available to authorized users.

## Background

Ovarian malignancies account for 4% of cancer in women and are the most frequent cause of death due to gynecological cancer in Western countries [1]. Carcinomas are the most common subtype, with the serous histotype being particularly prevalent [1]. Most serous ovarian carcinomas are genomically unstable. Approximately 50% of the tumors have defects in the homologous recombination DNA repair pathway, with *BRCA1* and *BRCA2* alterations as the most frequent, while the remaining half show less characteristic aberration patterns [2–4].

Genomic imbalances are widespread in serous ovarian carcinomas and in a large The Cancer Genome Atlas (TCGA) study of ovarian carcinomas, 113 significant focal DNA copy number alterations were identified [4]. Structural chromosomal aberrations are also frequently seen, with involvement of chromosome 19 being particularly common [4–10]. Chromosome 11 has been reported to be one of the recurrent partners in such rearrangements [9, 11, 12]. The functional consequences of these aberrations are not understood.

Genomic rearrangements may lead to transcriptional deregulation, gene truncations, or gene fusions that encode fusion proteins [13]. Abnormal transcription events can also result in chimeric RNAs, e.g., by RNA polymerase read-through of adjacent genes [14]. Fusion genes have been identified in several epithelial cancers [15], but none have so far been validated as recurrent in independent cohorts of ovarian cancer [13]. In a recent study of 92 serous ovarian carcinomas using DNA and RNA sequencing, gene breakage was found to be a common mechanism for inactivating tumor suppressor genes but no fusion gene was identified as a recurrent, biologically plausible driver in tumorigenesis [10]. Still, for subgroups of patients, gene fusions might play such a role.

In the present study, we looked for fusion gene candidates and aberrantly expressed genes as well as the possible mechanisms behind such expression changes in a series of ovarian carcinomas with cytogenetically identified chromosome 19 changes. We used two different genome-scale approaches: exon-level microarrays and transcriptome sequencing (RNA-seq).

## Methods

### Patient samples

Ovarian carcinomas from 16 women were included in the study. Three patients had bilateral tumors so that in total 19 carcinoma samples were examined (see Table 1 for clinical information). Histologic classification of the carcinomas identified 18 tumors as serous and one as undifferentiated (immunohistochemistry was negative for WT-1). The tumors were selected based on the presence of cytogenetically visible genomic changes involving chromosome 19; all tumors either had a structurally rearranged chromosome 19 in the karyotype or were the contralateral counterpart of a tumor with such a change. Material from the same patients has previously been examined by karyotyping, fluorescence in situ hybridization (FISH), and comparative genomic hybridization (CGH) analysis at the chromosomal level as well as by microarrays [16–18]. The tumor biobank has been registered according to national legislation and the study has been approved by the Regional Committee for Medical Research Ethics South-East, REK; project numbers S-07194a and 2.2007.425.

**Table 1:** Clinical information

Sample	Patient	Age	Dx	Stadium	Karyotype: chr19	RNA-seq	Neo chemo
1	I	66	HGSC	IIIC	No der(19)^b^		
2	II	47	HGSC	IIIB	der(19)	V	
3	IIIa	54	HGSC	IIIC	No der(19)^b^		
4	IIIb	54	HGSC	IIIC	der(19)	V	
5	IVa	61	HGSC	IIIC	Culture failure^b^		
6	IVb	61	HGSC	IIIC	der(19)	V	
7	V	52	HGSC	IIIC	der(19)	V	
8	VI	50	HGSC	IV	der(19)	V	
9	VII	66	HGSC	IV	der(19)	V	
10	VIII	59	HGSC	IV	der(19)	V	yes
11	IX	56	HGSC	IIIC	der(19)	V	
12	X	51	UC	IIC	der(19)	V	
13	XI	74	HGSC	IIIC	der(19)	V	yes
14	XII	57	HGSC	IIIC	46,XX^b^		
15	XIIIa	64	HGSC	IIC	der(19)	V	
16	XIIIb	64	HGSC	IIC	46,XX^b^		
17	XIV	63	HGSC	IIIC	der(19)	V	
18	XV	56	SC^a^	IIIC	der(19)		yes
19	XVI	77	HGSC	IIIC	der(19)	V	

*Dx* diagnosis, *HGSC* high-grade serous carcinoma, *Neo chemo* neoadjuvant chemotherapy, *UC* undifferentiated carcinoma

^a^SC = serous carcinoma, cannot be graded due to chemo

^b^bilateral tumor has der(19) in the karyotype

### Isolation of RNA

Fresh tumor tissue was frozen and stored at −80 °C. Total RNA was extracted using TRIzol reagent (Invitrogen, Carlsbad, CA) and miRNAeasy spin columns (Qiagen GmbH, Hilden, Germany). First, tumor tissue was homogenized in TRIzol and the aqueous phase was removed and used further with the Qiagen miRNeasy Mini kit according to the manufacturer’s protocol. Quantitation and quality control of the isolated total RNA were performed using NanoVue spectrophotometer (GE Healthcare, Little Chalfont, UK) and the Experion automated electrophoresis system (Bio-Rad Laboratories, Hercules, CA, USA). Total RNA degradation was evaluated by reviewing the electropherograms and the RNA quality indicator (RQI).

### RNA-seq

High-throughput paired-end RNA-sequencing was performed according to the TruSeq paired-end RNA-sequencing protocols from Illumina for Solexa sequencing on a Genome Analyzer IIx with paired-end module (Illumina Inc., San Diego, CA, USA) as described earlier [19]. Nucleotides of 76 base pair length were sequenced. The RNA-seq files gave on average 32 million read pairs per sample (range: 24–42 million). The FASTQC software was used for quality control of the raw sequence data [20]. The software FusionMap [21] (release date 2012–04-16; paired-end utility using default parameters) and the associated pre-built Human B37 and RefGene from the FusionMap website were used for discovery of fusion transcripts [22].

### RT-PCR and Sanger sequencing

Putative gene fusions were validated by Reverse Transcriptase-Polymerase Chain Reaction (RT-PCR) followed by Sanger sequencing. Expression of the housekeeping gene *ABL1* was used as internal control. cDNA (originally prepared for the microarray analysis, see below) equivalent to 10 ng RNA was amplified using TAKARA Premix Ex Taq (TaKaRa-Bio, Europe/SAS, Saint-Germain-en-Laye, France) with PCR conditions as previously described [23]. Primers are listed in Additional file 1: Table S1. PCR products were analyzed by electrophoresis through 1.0% agarose gel and products of the correct length were subjected to Sanger sequencing using BigDye Terminator V1.1 cycle sequencing kit on an ABI 3500 Genetic Analyzer (ThermoFisher Scientific, Waltham, MA, USA). The BLAST and BLAT programs were used for computer analysis of sequence data [24, 25].

### Microarray gene expression analysis

100 ng total RNA was used as input for global gene expression analysis at the exon level using Affymetrix GeneChip Human Exon 1.0 ST Arrays (Affymetrix, Santa Clara, CA, USA). Each microarray contained 1.4 million probe sets (the majority of which were comprised of four probes), where each probe set corresponded to approximately one known or computationally predicted exon. RNA from each sample was individually amplified, reversely transcribed, fragmented, and labeled. Labeled sense strand cDNA was hybridized onto the arrays for 16–18 h, after which the arrays were washed, stained, and scanned as described earlier [26].

Cell intensity (CEL)-files from the tumor samples were background corrected, inter-chip quantile normalized, and summarized at gene level by the robust multi-array average (RMA) approach [27] implemented in the Affymetrix Expression Console 1.1 software. Genes, i.e., transcript clusters annotated with gene symbols, were identified using the HuEx-1_0-st-v2.r2 core library files and the annotation files HuEx-1_0-st-v2.na35.hg19.probeset.csv and HuEx-1_0-st-v2.na35.hg19.transcript.csv, available from the Affymetrix web page [28]. For technical control, three runs of one normal ovarian sample (Cat#HR-406 Human Ovary Total RNA, Zyagen, San Diego, CA, USA) were included.

### Identification of gene fusions from exon-microarray data

To identify putative fusion events from the microarray data, we computationally selected genes in which some of the samples showed outlier expression profiles for one of the gene moieties (i.e., either the 5′ or the 3′ end of the transcript), as previously described by Hoff et al. [29]. We particularly focused on fusions corresponding to structural changes of chromosome 19 and/or its partner chromosomes in the rearrangements previously identified by karyotyping [12]. For robustness, filtering procedures excluded breakpoint candidates with only one probe set on either side of the suggested breakpoint. Breakpoints in genes with gene symbols including two gene names (e.g. “DCAF8L2 // LOC101928481”) or “---” were also filtered out. Candidate fusion partner genes were ranked according to the magnitude of their expression deviation from the median of the set, and the results were compared with the RNA-seq data analysis.

## Results

We used two different approaches to identify aberrantly expressed genes possibly involved in fusion events: 1) a combination of karyotypic information and RNA-seq data; 2) a comparison of microarray and RNA-seq data.

### RNA-seq fusion candidates

RNA-seq data on 13 ovarian carcinomas were analyzed by the software FusionMap, yielding a list of 2069 unique fusion gene candidates from all the samples combined. The average number of fusion candidates per sample was 274 (range: 209–345).

Three tumors (samples 7, 11, and 17) had structural rearrangements involving chromosomes 11 and 19 in their karyotype, with breakpoint positions in 11q13 ~ q14, 19p13, and 19q13. Five fusion gene candidates were identified by RNA-seq analysis as corresponding to these breakpoints (Additional file 1: Table S2). In sample 7, there was a fusion sequence between Dipeptidyl-Peptidase 9 (*DPP9*, mapping to 19p13.3) and Protein Phosphatase 6, Regulatory Subunit 3 (*PPP6R3*, 11q13.2). The BLAT analysis of the *DPP9*-*PPP6R3* fusion transcript sequence showed 100% identity to only these two gene sequences, supporting the presence of a true fusion rearrangement (Additional file 1: Table S3). The remaining 11–19 fusion candidates did not show similar specificity by BLAT.

#### DPP9

The junction in the *DPP9-PPP6R3* fusion was found between exon 11 of *DPP9* (accession number NM_139159.4) and exon 18 of *PPP6R3* (NM_001164162.1).

**Fig. 1:**
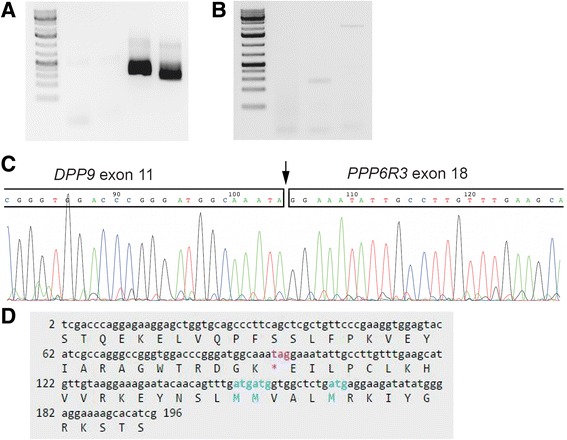
Experimental validation of *DPP9* fusions. **a** RT-PCR validation of the *DPP9*-*PPP6R3* rearrangement in sample 7. Lanes from left: (1) DNA size marker, (2–3) PCR-reactions, and (4–5) nested-PCR. **b** RT-PCR with primers of the putative *DPP9*-*PLIN3* rearrangement gave a weak band of the expected length of base pairs (3rd lane, band indicating PCR-product of approximately 300 base pairs). **c**-**d** Sanger sequencing. Partial sequence of the *DPP9-PPP6R3* rearrangement. The fusion junction is marked by an arrow. The rearrangement introduces a stop codon in the new sequence (“tag”, marked by an asterisk)

The presence of the *DPP9*-*PPP6R3* fusion was validated by RT-PCR and Sanger sequencing (Fig. 1). Sanger sequencing showed that the fusion introduced a stop codon directly after the junction (Fig. 1), indicating a truncated DPP9 protein, if any protein at all. Presence of the *DPP9-PPP6R3* fusion rearrangement was tested for by RT-PCR in the remaining samples with cDNA of sufficient quality (*n =* 15) but the hybrid transcript was not expressed by any other tumor.

**Fig. 2:**
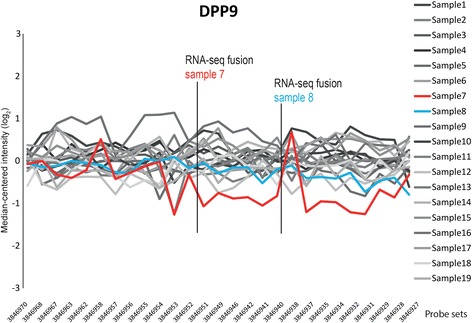
*DPP9* gene expression profile. Sample-wise median-centered exon-level expression of *DPP9* (y-axis; log2) is plotted for each of the probe sets (x-axis; sorted according to their genome positions). The *DPP9-PPP6R3* fusion rearrangement was identified in sample 7 (*red*) and the gene expression is reduced correspondingly, downstream of the RNA-seq fusion breakpoint in exon 11 (targeted by probe set 3,846,952). The *DPP9-PLIN3* rearrangement was identified in sample 8 (*blue*), and the expression is reduced downstream of the RNA-seq fusion breakpoint in exon 16 (probe set 3,846,940)

We then searched the RNA-seq data for other fusion transcripts involving *DPP9* and found it recombined with another partner, Perilipin 3 (*PLIN3*, NM_005817.4, 19p13.3), in sample 8 (Additional file 1: Table S2). The fusion junction detected by RNA seq was between exon 16 of *DPP9* (NM_139159.4) and exon 8 of *PLIN3*. The gene expression of *DPP9* in this sample showed a decrease in expression downstream of exon 16, however, the decrease in expression was not as marked as in sample 7 (Fig. 2). The expression of *PLIN3* was similar in all analyzed tumors, admittedly with the caveat that for this gene, the microarray analysis data was based on only one probe set so we cannot identify possible breakpoints (data not shown). The BLAT analysis of the *DPP9*-*PLIN3* fusion sequence showed 100% identity to only these two gene sequences (Additional file 1: Table S3). RT-PCR of the *DPP9-PLIN3* fusion gave a band on the agarose gel corresponding to the expected length (361 base pairs; Fig. 1), but we were not able to get an informative sequence from the product by Sanger sequencing. BLAT analysis of the junction sequence supported the presence of a gene fusion since the *DPP9-PLIN3* fusion sequence matched only these two gene sequences. The genomic region where both *DPP9* and *PLIN3* reside, i.e., cytoband 19p13, was found to be rearranged in the sample with the reported fusion (case 8) inasmuch as the karyotype showed a der(19)add(19)(p13)add(19)(q13).

### Exon-level gene expression

**Fig. 3:**
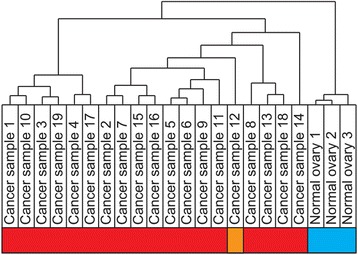
Hierarchical clustering. Hierarchical clustering of the 22 expression microarray runs (19 tumors and 1 normal control run in triplicate) based on the total data set at gene-level. Normal runs (*blue*) separate from cancer samples; the undifferentiated carcinoma (*orange*) clusters together with the serous carcinomas (*red*)

Microarray expression analysis provided information on 17,506 genes (i.e., unique transcript clusters annotated with gene symbols) at exon-level and 17,638 genes at gene-level. As expected, hierarchical clustering of the global expression data set at gene-level separated cancer samples from controls (Fig. 3). The undifferentiated carcinoma (cancer sample 12) clustered together with the serous carcinomas.

**Fig. 4:**
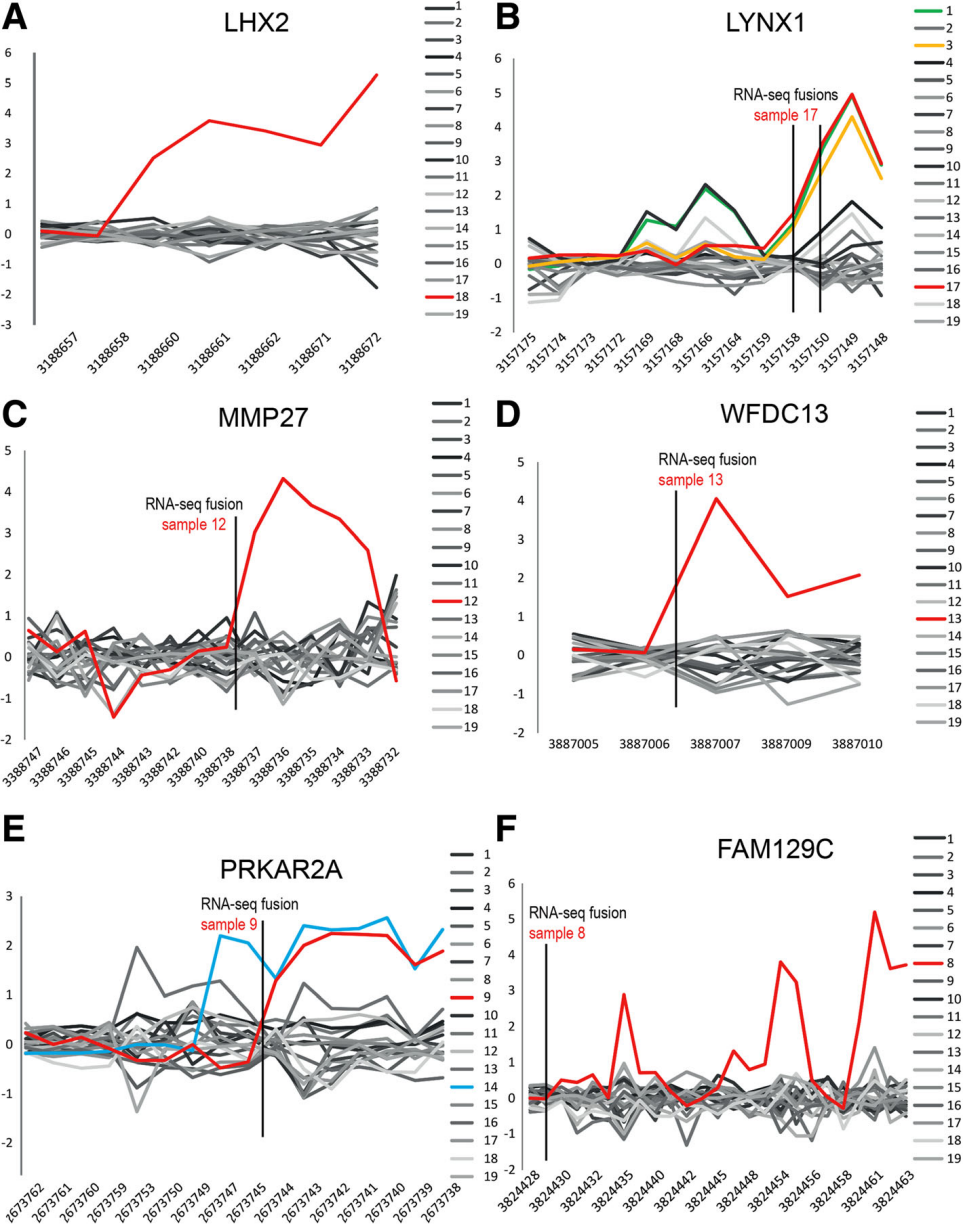
Exon-level gene expression profiles with RNA-seq fusion breakpoints. The Y-axes show the median-centered gene expression values which are in log_2_, X-axes show the probe sets sorted according to their genome positions. **a** For *LHX2*, sample 18 (*red*) has clearly deviating expression from the rest of the samples in the 3′ end. **b** Expression of *LYNX1*. For sample 17, RNA-seq analyses nominated both the fusion *FCF1*-*LYNX1* and *LYNX1*-*FCF1*. Thus, two RNA-seq fusion breakpoints are indicated. **c** Expression of *MMP27*. **d** Expression of *WFDC13*. **e** Expression of *PRKAR2A*. (F) Expression of *FAM129C*

We used the exon-level microarray data as a screening tool to search for genes in which either the 5′ or 3′ end expression differed from the median of the sample set, i.e., showed “transcript breakpoints”, hypothesizing that transcribed fusion rearrangements would rank high on this list. In the total data set, the algorithm identified 120,099 such breakpoints after filtering procedures. The top 1000 candidates, corresponding to approximately 0.8% of the suggested breakpoints, were selected for further analysis (Table 2: Exon-level expression analysis). A clearly aberrant expression was found for the LIM homeobox 2 gene (*LHX2*, XM_006717323, 9q33.3) in sample 18 (Fig. 4a), with a breakpoint ranking as the 15th best of all breakpoints. In *LHX2*, the microarray results showed a higher level of expression from the probe set corresponding to exon 2 (i.e., probe set 3,188,660). This was not among the samples from which RNA-seq data were available (Table 1); however, we searched for involvement of this gene in fusions in the RNA-sequenced samples but found no fusion gene candidates involving *LHX2*.

**Table 2:** Exon-level expression analysis

	Total gene set	Chr 19 genes
No. breakpoints analyzed	1000	28
Unique genes	706	25
Recurrent genes	176	2
RNA-seq fusion candidates	86 genes	4
Exon breakpoint + RNA-seq fusion	35^a^	1

^a^In 35 events, the same gene was among the top 1000 transcript-breakpoints and a nominated RNA-seq fusion gene partner in the same sample. Two genes are listed more than once: *TAP2* and *COL9A1*, which are listed two and three times, respectively. Table S4 lists the RNA-seq fusion gene candidates of the 32 genes

The microarray data and the RNA-seq data were combined by comparing the top 1000 exon-level breakpoints to the complete list of RNA-seq fusion candidates. Two genes, the Transporter 2, ATP-Binding Cassette, Sub-Family B (*TAP2*) and the Collagen, Type IX, Alpha 1 (*COL9A1*), were found with altered expression in two and three of the samples, respectively. Thus, a total of 32 genes showed a match between presence of altered expression on microarray and involvement as an RNA-seq fusion transcript candidate (Additional file 1: Table S4).

Among the genes in which changes in the expression level fit with a possible fusion detected by transcriptome sequencing, we found the following:

#### LYNX1

Ly6/Neurotoxin 1 (*LYNX1*, NM_177458.1, 8q24.3) was found as the highest ranked gene. In sample 17, its expression increased markedly from probe set 3,157,159 and on (Fig. 4b). The nominated RNA-seq 5′ fusion partner was FCF1 RRNA-Processing Protein (*FCF1*, 14q24.3, NM_015962; Additional file 2: Figure S1). In two additional instances (samples 1 and 3), the gene expression profiles for *LYNX1* were similar (see Fig. 4b); however, these two tumors were not RNA-sequenced. Analyses of RNA-seq data also identified *LYNX1* as the 3′ gene in the reciprocal fusion in the same sample, i.e., *LYNX1*-*FCF1*. The two fusion junction transcripts from the RNA-seq data both mapped to several genomic locations according to BLAT analysis.

#### MMP27

A transcript breakpoint in the Matrix Metallopeptidase 27 gene (*MMP27*, NM_022122, 11q22.2) in sample 12 was also highly ranked, i.e., breakpoint number 80 (Additional file 1: Table S3). The RNA-seq showed a matching fusion with Transmembrane Protein 123 (*TMEM123*, NM_052932, 11q22.2; Additional file 2: Figure S1). The junction in the *TMEM123*-*MMP27* transcript was found between exons 2 and 7 targeted by the probe sets 3,388,639 and 3,388,742, respectively (Fig. 4c, Additional file 2: Figure S1). In this fusion, the *TMEM123* 5’gene was more strongly expressed than the 3’gene. The two genes reside only 220 kb apart on the same strand with two other matrix metallopeptidase genes in-between (*MMP7* and *MMP20*).

#### WFDC13

The gene WAP Four-Disulfide Core Domain 13 (*WFDC13*, NM_172005, 20q13.12) had an RNA-seq suggested fusion after exon 2, matching the point of increase in gene expression (Fig. 4d). The putative fusion partner was the Zinc Finger And BTB Domain Containing 46 (*ZBTB46*, 20q13.33, NM_025224, Additional file 2: Figure S1) which resides 18 M base pairs (Mbp) downstream, leading to formation of a *ZBTB46*-*WFDC13* transcript.

#### PRKAR2A

The Protein Kinase, CAMP-Dependent, Regulatory, Type II, Alpha gene (*PRKAR2A*, NM_004157, 3p21.31) showed altered expression from exon 7 on in sample 9 (Fig. 4e). The RNA-seq data identified the Plexin B1 gene (*PLXNB1*, NM_002673.5, 3p21.31, Additional file 2: Figure S1), which is located 347 k base pairs (kbp) downstream of *PRKAR2A,* as a putative partner with three different fusion junction sequences (Additional file 1: Table S4), of which two showed 100% identity according to BLAT analysis. The *PRKAR2A* gene showed altered expression in an additional tumor (sample 14) with increase from exon 9 on. Unfortunately, the latter sample was not RNA-sequenced.

#### Chromosome 19 sub-analysis

We also performed a separate analysis of genes from chromosome 19 that were among the top ranked breakpoint candidates (Table 2). The highest ranked exon-level breakpoint in a gene from chromosome 19 was found for the Zinc Finger Protein 257 gene (*ZNF257*) in sample 15. This gene ranked as number 30 in the total data set. Analysis of the RNA-seq data gave no corresponding fusion partner information.

When collating the lists of transcript breakpoints and RNA-seq fusion transcript candidates, the highest ranked chromosome 19 gene was seen to be Family With Sequence Similarity 129, Member C (*FAM129C*, NM_173544.4, 19p13.11) with breakpoint number 37 in the total data set (Fig. 4f). In sample 8, the RNA-seq analysis showed involvement of this gene in a fusion with the DET1 and DDB1 Associated 1 gene (*DDA1*, NM_024050, 19p13.11, Additional file 2: Figure S1). *DDA1*-*FAM129C* was furthermore among the RNA-seq candidates that were seen to correspond to a karyotypic finding – this sample had an identified breakpoint in 19p13 – so for this fusion candidate examination at all three resolution levels led to concordant results.

## Discussion

Large cohort studies of serous ovarian carcinomas have not found recurrent gene fusions [4, 10]. However, isolated events in single patients can still be important steps in tumorigenesis and/or progression of individual tumors.

We report here a combination of transcriptome analyses of ovarian carcinomas selected on the basis of known presence of structural rearrangements of chromosome 19 in their karyotypes. The *DPP9*-*PPP6R3* fusion rearrangement was identified by RNA-seq and exon-level microarray analyses and validated by RT-PCR in one ovarian serous carcinoma. The fusion leads to disruption and subsequent deregulation of *DPP9* gene expression. *DPP9* was found rearranged with *PLIN3* in another serous carcinoma, and also in this case the *DPP9* expression was disrupted and lowered toward the 3′ end. The lost fragment of the *DPP9* transcript includes the code for the functional peptidase and esterase-lipase domains of the DPP9 protein. Previously, Hoogstraat et al. [30] found rearrangement of *DPP9* in a high-grade serous ovarian carcinoma by means of whole-genome mate-pair sequencing, a *DPP9*-*PAX2* in-frame rearrangement with the breakpoint after exon 11 in *DPP9*, i.e., close to the breakpoint identified in the present *DPP9*-*PPP6R3* fusion. No information was reported on *DPP9* expression. Despite the presence of three different partner genes (*PPP6R3*, *PLIN3,* and *PAX2*) involved in the *DPP9-* rearrangements seen until now, it seems that they all lead to loss of the 3′ part of the *DPP9* transcript (Additional file 1: Table S2). The effect of the disruption of *DPP9* expression was evaluated by BLAT; despite different fusion breakpoint positions, both the *DPP9*-*PPP6R3* and the *DPP9*-*PLIN3* rearrangements would lead to loss of the same functional domains at protein level, namely the peptidase and esterase-lipase domains.

In the TCGA dataset of high-grade serous ovarian carcinomas, 7% of the samples had downregulated gene expression of *DPP9* without DNA copy number alteration (i.e., 23 of 316 samples, data available at cBio Cancer Genomics Portal, Memorial Sloan-Kettering Cancer Center (MSKCC), www.cbioportal.org, default settings [4, 31, 32]). This was also the case in our samples where *DPP9* was found downregulated without DNA copy number change. Patch et al. [10] showed that, in ovarian carcinomas, gene breakage was a common mechanism for inactivating tumor suppressor genes (*RB1*, *NF1*, *RAD51B*, and *PTEN*); thus, loss-of-function gene changes could explain the observed *DPP9* downregulation.

We have found no studies describing fusions involving *DPP9* in the Mitelman database or in the TCGA fusion gene portal [33, 34]; thus, this is the first report showing that *DPP9*-rearrangements occur in serous ovarian carcinoma.

The *DPP9* gene encodes a serine protease that belongs to the DPPIV subfamily and is ubiquitously expressed (www.uniprot.org, [35]). Proteases may act as tumor suppressors [36] as exemplified by *DPP4* which is homologous to *DPP9,* both encode proteins that harbor the DPPIV-domain as well as hydrolase and peptidase conserved domains. Loss of *DPP4* contributes to tumorigenesis in several cancers [36], including ovarian carcinoma [37], whereas forced expression has shown growth inhibitory effect on glioma cells [38]. The DPP9 protein participates in cell signaling and has several tumor suppressing abilities such as inducing apoptosis, suppressing proliferation, and attenuating activation of the oncogene AKT (protein kinase B) [39]. The *DPP9-*rearrangements resulted in loss of the active sites of DPP9; it is well known that gene fusions may represent loss-of-function events which play a role in carcinogenesis, as reported in colorectal [40] and prostate cancer [41].

*LHX2* was among the genes showing differential expression of either the 5′ or 3′ end of the transcript (Fig. 4a). Different gene expression profiles among samples can sometimes be due to expression of alternative transcripts. This does not seem to be the case for *LHX2*, however, which has several annotated transcript isoforms according to the ENSEMBL data base [42], but none that can explain the observed gene expression breakpoint*.* The gene encodes a transcriptional activator that has been reported to promote tumor growth and metastasis in breast cancer [43]. Its involvement in three gene fusions (*IGH-LHX2*, *ADAMTS13-LHX2*, and *AAK1-LHX2*) has been described in chronic myelogenous leukemia, breast cancer, and uterine carcinosarcoma, respectively [34, 44].

Three of our samples showed strong expression of the 3′ of *LYNX1*. The exon-level gene expression results could possibly be explained by alternative transcripts since the increased expression matches the starting point of two transcript isoforms (transcripts ENST00000521396 and ENST00000317543). No fusions involving *LYNX1* have been reported in the literature so far, but the suggested fusion partner *FCF1* was involved in a *TIMM9*-*FCF1* fusion in an astrocytoma [34]. Another example of a transcript breakpoint that corresponds to the start of a transcript isoform is provided by *PRKAR2A*, but in that case a true fusion event seems more likely given the RNA-seq data. A *CDC25A*-*PRKAR2A* fusion was previously reported in an ovarian carcinoma [34].

Deregulation of *MMP27* was consonant with the findings made by cytogenetics-based genomic analysis, RNA-sequencing, and studies of the exon-level gene expression profile. The RNA-seq listed *TMEM123* as the 5’partner. A fusion of the same two genes has been reported in a breast carcinoma, while in one ovarian carcinoma *TMEM123* was found fused with *MMP7* [34]. In all these three reports, *TMEM123* had the same breakpoint position. Since *MMP27* and *TMEM123* map closely on the same DNA strand (in 11q22.2), an interstitial deletion could have caused the fusion, and imbalances in this chromosomal region have been seen by both HR-CGH and karyotyping [12]. Upregulation of matrix metalloproteinases are known to play a role in cancer and metastasis [45].

Previously performed karyotypic analyses gave information about the possible chromosomal rearrangements behind several of the identified fusion gene candidates. One example is *ZBTB46*-*WFDC13*, where both genes map to chromosome 20 and where the karyotype included the following highly rearranged chromosome: der(19)(20p13 → 20q13::19p13 → 19q13::8q22 → 8qter) [18]; it is reasonable to assume that additional submicroscopic alterations were also present leading to the gene rearrangement. Interestingly, *WFDC13* is closely related to the clinically important gene *WFDC2* which is also known as Human Epididymis Protein 4. *WFDC2* is known to be overexpressed in ovarian carcinomas and encodes the HE4 protein, one of very few biomarkers used to monitor the disease in ovarian cancer patients [46].

*PRKAR2A*-rearrangements have been reported as an in-frame *CDC25A*-*PRKAR2A* fusion in the TCGA ovarian cancer data set [34]. Interestingly, the breakpoint thus identified corresponds to the exon-level breakpoint in our sample 14. This sample was not RNA-sequenced, so we do not know if this rearrangement caused the gene expression profile.

The chromosome 19 sub-analysis highlighted the *FAM129C* gene which by RNA-seq was found to participate in the generation of the fusion gene candidate *DDA1*-*FAM129C*. Both the genomic findings and gene expression analyses gave results pointing in the same direction. The two genes are located only 211 kbp apart on the same strand (19p13.11); thus, an interstitial deletion could have caused the fusion. Another fusion involving this gene, namely the *CLTC-FAM129C* in breast cancer, was reported before [34]. Little is known about the function of *FAM129C* or the possible consequences of its upregulated expression.

Some notes of caution are warranted on the limitations of the approach we have used to identify potential fusion gene partners by looking for genes with different expression of their 3′ and 5′ ends. Gene fusions involving the promoter region of one gene and the coding sequence of another will not be detected and would constitute “false negatives”. Furthermore, the algorithm might misevaluate expression of different transcript isoforms from a single gene and nominate it as a fusion gene candidate, i.e., a “false positive”. The algorithm is also sensitive to technical noise in the data. Some of the breakpoints identified did not seem to be due to abnormal transcription but to noise within a limited sample set. By using different methods for gene expression analysis and comparing the results, we have tried to identify gene expression alterations that arise due to fusion events rather than due to expression of different transcript isoforms or alternative splicing.

## Conclusions

By combining karyotype information, RNA-seq, and exon-level gene expression microarray analysis, we have identified several fusion gene candidates in high-grade serous ovarian carcinomas. Most of these fusions seem to be isolated events. However, rearrangements of *DPP9*, leading to decreased expression of its 3′ end, were identified in two cases and might possibly result in loss of tumor suppressor function.

## Additional files

Additional file 1: Table S1. RT-PCR primers. **Table S2.** RNA-seq fusion gene candidates. **Table S3.** BLAT results of fusion transcripts. **Table S4.** RNA-seq fusion gene partner candidates among the top 1000 exon-level expression breakpoints. (XLS 86 kb)

Additional file 2 **Figure S1.** Gene expression of the nominated fusion partners. In general, the expression of the 5′ genes in the samples with the reported fusion genes does not differ from the rest. Median-centered gene expression values are in log_2_ (y-axes). Probe sets are shown along the x-axis, sorted according to their genome positions. Examples of fusion gene candidates: (A) *DPP9*-*PPP6R3*; (B) *FCF1*-*LYNX1*; (C) *TMEM123*-*MMP27*; (D) *ZBTB46*- *WFDC13*; (E) *PLXNB1*-*PRKAR2A*; and (F) *DDA1*-*FAM129C*.

## Abbreviations

CEL: Cell intensity
CGH: Comparative genomic hybridization
FISH: Fluorescence in situ hybridization
RMA: Robust multi-array average
RNA-seq: RNA sequencing
RQI: RNA quality indicator
RT-PCR: Reverse Transcriptase-Polymerase Chain Reaction
TCGA: The Cancer Genome Atlas

## References

[1] Kurman RJ, Hendrick Ellenson L, Ronnett BM (2011). Blaustein’s pathology of the female genital tract.

[2] Bowtell DD, Bohm S, Ahmed AA, Aspuria P-J, Bast RC, Beral V, Berek JS, Birrer MJ, Blagden S, Bookman MA (2015). Rethinking ovarian cancer II: reducing mortality from high-grade serous ovarian cancer. Nat Rev Cancer.

[3] Ciriello G, Miller ML, Aksoy BA, Senbabaoglu Y, Schultz N, Sander C (2013). Emerging landscape of oncogenic signatures across human cancers. Nat Genet.

[4] Cancer Genome Atlas Research Network (2011). Integrated genomic analyses of ovarian carcinoma. Nature.

[5] Pejovic T, Heim S, Mandahl N, Baldetorp B, Elmfors B, Floderus UM, Furgyik S, Helm G, Himmelmann A, Willen H (1992). Chromosome aberrations in 35 primary ovarian carcinomas. Genes Chromosomes Cancer..

[6] Pejovic T, Heim S, Mandahl N, Elmfors B, Floderus UM, Furgyik S, Helm G, Willen H, Mitelman F (1989). Consistent occurrence of a 19p+ marker chromosome and loss of 11p material in ovarian seropapillary cystadenocarcinomas. Genes Chromosomes Cancer..

[7] Taetle R, Aickin M, Yang JM, Panda L, Emerson J, Roe D, Adair L, Thompson F, Liu Y, Wisner L (1999). Chromosome abnormalities in ovarian adenocarcinoma: I. Nonrandom chromosome abnormalities from 244 cases. Genes Chromosomes Cancer..

[8] Kiechle-Schwarz M, Bauknecht T, Schmidt J, Walz L, Pfleiderer A (1995). Recurrent cytogenetic aberrations in human ovarian carcinomas. Cancer Detect Prev.

[9] Heim S, Mitelman F (2015). Cancer Cytogenetics: chromosomal and molecular genetic aberrations of tumor cells.

[10] Patch A-M, Christie EL, Etemadmoghadam D, Garsed DW, George J, Fereday S, Nones K, Cowin P, Alsop K, Bailey PJ (2015). Whole–genome characterization of chemoresistant ovarian cancer. Nature.

[11] Kiechle-Schwarz M, Bauknecht T, Karck U, Kommoss F, du Bois A, Pfleiderer A (1994). Recurrent cytogenetic aberrations and loss of constitutional heterozygosity in ovarian carcinomas. Gynecol Oncol.

[12] Micci F, Weimer J, Haugom L, Skotheim RI, Grunewald R, Abeler VM, Silins I, Lothe RA, Trope CG, Arnold N (2009). Reverse painting of microdissected chromosome 19 markers in ovarian carcinoma identifies a complex rearrangement map. Genes Chromosomes Cancer..

[13] Mertens F, Johansson B, Fioretos T, Mitelman F (2015). The emerging complexity of gene fusions in cancer. Nat Rev Cancer.

[14] Annala MJ, Parker BC, Zhang W, Nykter M (2013). Fusion genes and their discovery using high throughput sequencing. Cancer Lett.

[15] Kumar-Sinha C, Kalyana-Sundaram S, Chinnaiyan A (2015). Landscape of gene fusions in epithelial cancers: seq and ye shall find. Genome Medicine.

[16] Micci F, Haugom L, Abeler VM, Davidson B, Trope CG, Heim S (2014). Genomic profile of ovarian carcinomas. BMC Cancer.

[17] Micci F, Haugom L, Ahlquist T, Abeler VM, Trope CG, Lothe RA, Heim S (2010). Tumor spreading to the contralateral ovary in bilateral ovarian carcinoma is a late event in clonal evolution. J Oncology.

[18] Micci F, Skotheim RI, Haugom L, Weimer J, Eibak AM, Abeler VM, Trope CG, Arnold N, Lothe RA, Heim S (2010). Array-CGH analysis of microdissected chromosome 19 markers in ovarian carcinoma identifies candidate target genes. Genes Chromosomes Cancer..

[19] Nyquist KB, Panagopoulos I, Thorsen J, Haugom L, Gorunova L, Bjerkehagen B, Fossa A, Guriby M, Nome T, Lothe RA (2012). Whole-transcriptome sequencing identifies novel IRF2BP2-CDX1 fusion gene brought about by translocation t(1;5)(q42;q32) in mesenchymal chondrosarcoma. PLoS One.

[20] FASTQ. Babraham Bioinformatics. http://www.bioinformatics.babraham.ac.uk/projects/fastqc. Accessed 8 Apr 2016.

[21] Ge H, Liu K, Juan T, Fang F, Newman M, Hoeck W (2011). FusionMap: detecting fusion genes from next-generation sequencing data at base-pair resolution. Bioinformatics.

[22] FusionMap website: http://www.omicsoft.com/fusionmap/ Accessed 8 Apr 2016.

[23] Agostini A, Panagopoulos I, Andersen HK, Johannesen LE, Davidson B, Trope CG, Heim S, Micci F (2015). HMGA2 expression pattern and TERT mutations in tumors of the vulva. Oncol Rep.

[24] BLAST. http://blast.ncbi.nlm.nih.gov/Blast.cgi. Accessed 8 Apr 2016.

[25] BLAT. http://genome.ucsc.edu/cgi-bin/hgBlat. Accessed 8 Apr 2016.

[26] Smebye ML, Sveen A, Haugom L, Davidson B, Trope CG, Lothe RA, Heim S, Skotheim RI, Micci F (2014). Chromosome 19 rearrangements in ovarian carcinomas: zinc finger genes are particularly targeted. Genes Chromosomes Cancer.

[27] Irizarry RA, Hobbs B, Collin F, Beazer-Barclay YD, Antonellis KJ, Scherf U, Speed TP (2003). Exploration, normalization, and summaries of high density oligonucleotide array probe level data. Biostatistics.

[28] Affymetrix. http://www.affymetrix.com/estore/catalog/131452/AFFY/Human+Exon+ST+Array#1_1. Accessed 21 June 2016.

[29] Hoff AM, Johannessen B, Alagaratnam S, Zhao S, Nome T, Lovf M, Bakken AC, Hektoen M, Sveen A, Lothe RA (2015). Novel RNA variants in colorectal cancers. Oncotarget.

[30] Hoogstraat M, de Pagter MS, Cirkel GA, van Roosmalen MJ, Harkins TT, Duran K, Kreeftmeijer J, Renkens I, Witteveen PO, Lee CC (2014). Genomic and transcriptomic plasticity in treatment-naive ovarian cancer. Genome Res.

[31] Cerami E, Gao J, Dogrusoz U, Gross BE, Sumer SO, Aksoy BA, Jacobsen A, Byrne CJ, Heuer ML, Larsson E (2012). The cBio cancer genomics portal: an open platform for exploring multidimensional cancer genomics data. Cancer Discovery.

[32] Gao J, Aksoy BA, Dogrusoz U, Dresdner G, Gross B, Sumer SO, Sun Y, Jacobsen A, Sinha R, Larsson E (2013). Integrative analysis of complex cancer genomics and clinical profiles using the cBioPortal. Sci Signal.

[33] Mitelman F, Johansson B, Mertens F: Mitelman database of chromosome aberrations and gene fusions in cancer, http://cgap.nci.nih.gov/Chromosomes/Mitelman. Accessed 21 June 2016.

[34] Yoshihara K, Wang Q, Torres-Garcia W, Zheng S, Vegesna R, Kim H, Verhaak RG (2015). The landscape and therapeutic relevance of cancer-associated transcript fusions. Oncogene.

[35] Consortium TU (2015). UniProt: a hub for protein information. Nucleic Acids Res.

[36] Lopez-Otin C, Matrisian LM (2007). Emerging roles of proteases in tumour suppression. Nat Rev Cancer.

[37] Kajiyama H, Kikkawa F, Suzuki T, Shibata K, Ino K, Mizutani S (2002). Prolonged survival and decreased invasive activity attributable to dipeptidyl peptidase IV overexpression in ovarian carcinoma. Cancer Res.

[38] Busek P, Stremenova J, Sromova L, Hilser M, Balaziova E, Kosek D, Trylcova J, Strnad H, Krepela E, Sedo A (2012). Dipeptidyl peptidase-IV inhibits glioma cell growth independent of its enzymatic activity. Int J Biochem Cell Biol.

[39] Yao TW, Kim WS, Yu DM, Sharbeen G, McCaughan GW, Choi KY, Xia P, Gorrell MD (2011). A novel role of dipeptidyl peptidase 9 in epidermal growth factor signaling. Molecular Cancer Res.

[40] Yu J, Wu WKK, Liang Q, Zhang N, He J, Li X, Zhang X, Xu L, Chan MTV, Ng SSM (2016). Disruption of NCOA2 by recurrent fusion with LACTB2 in colorectal cancer. Oncogene.

[41] Berger MF, Lawrence MS, Demichelis F, Drier Y, Cibulskis K, Sivachenko AY, Sboner A, Esgueva R, Pflueger D, Sougnez C (2011). The genomic complexity of primary human prostate cancer. Nature.

[42] Cunningham F, Amode MR, Barrell D, Beal K, Billis K, Brent S, Carvalho-Silva D, Clapham P, Coates G, Fitzgerald S (2015). Ensembl 2015. Nucleic Acids Res.

[43] Kuzmanov A, Hopfer U, Marti P, Meyer-Schaller N, Yilmaz M, Christofori G (2014). LIM-homeobox gene 2 promotes tumor growth and metastasis by inducing autocrine and paracrine PDGF-B signaling. Mol Oncol.

[44] Nadal N, Chapiro E, Flandrin-Gresta P, Thouvenin S, Vasselon C, Beldjord K, Fenneteau O, Bernard O, Campos L, Nguyen-Khac F (2012). LHX2 deregulation by juxtaposition with the IGH locus in a pediatric case of chronic myeloid leukemia in B-cell lymphoid blast crisis. Leuk Res.

[45] Egeblad M, Werb Z (2002). New functions for the matrix metalloproteinases in cancer progression. Nat Rev Cancer.

[46] Drapkin R, von Horsten HH, Lin Y, Mok SC, Crum CP, Welch WR, Hecht JL (2005). Human epididymis protein 4 (HE4) is a secreted glycoprotein that is overexpressed by serous and endometrioid ovarian carcinomas. Cancer Res.

