# Supplementary figures and images for: Involvement of DPP9 in gene fusions in serous ovarian carcinoma
Source: BMC Cancer. 2017 Sep 11;17:642. doi: 10.1186/s12885-017-3625-6 (PMC5594496; doi:10.1186/s12885-017-3625-6)

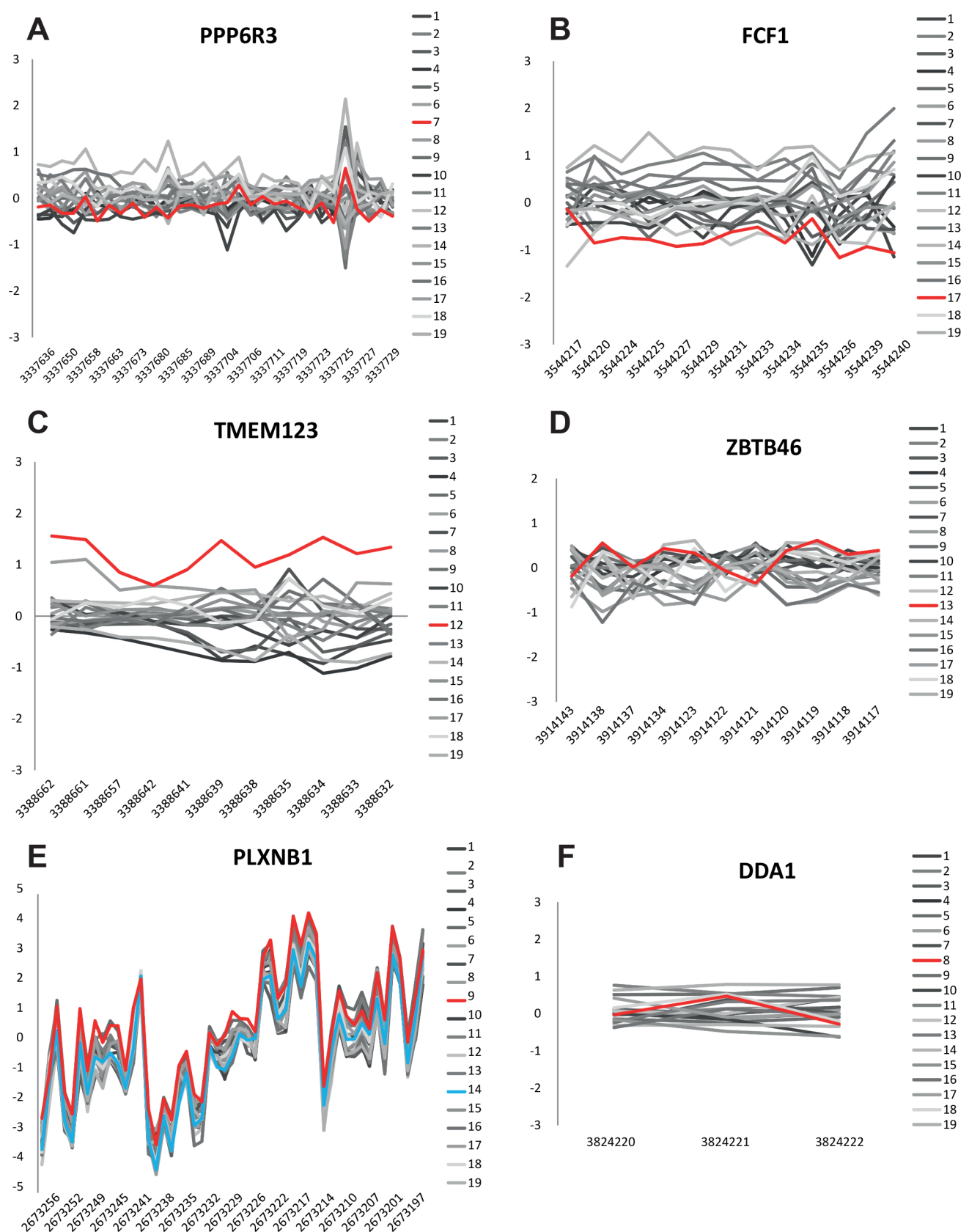

Supplement: Supplementary file 2 — Figure S1. Gene expression of the nominated fusion partners. In general, the expression of the 5′ genes in the samples with the reported fusion genes does not differ from the rest. Median-centered gene expression values are in log2 (y-axes). Probe sets are shown along the x-axis, sorted according to their genome positions. Examples of fusion gene candidates: (A) DPP9-PPP6R3; (B) FCF1-LYNX1; (C) TMEM123-MMP27; (D) ZBTB46- WFDC13; (E) PLXNB1-PRKAR2A; and (F) DDA1-FAM129C. [file 12885_2017_3625_MOESM2_ESM.pdf]
